# Supplementary material for: Effects of Scutellaria baicalensis Extract-Induced Exosomes on the Periodontal Stem Cells and Immune Cells under Fine Dust
Source: Nanomaterials (Basel). 2024 Aug 27;14(17):1396. doi: 10.3390/nano14171396 (PMC11397387; doi:10.3390/nano14171396)
Supplement: Supplementary file 1 [file nanomaterials-14-01396-s001.zip › nanomaterials-3151286-supplementary.pdf]

Full gels

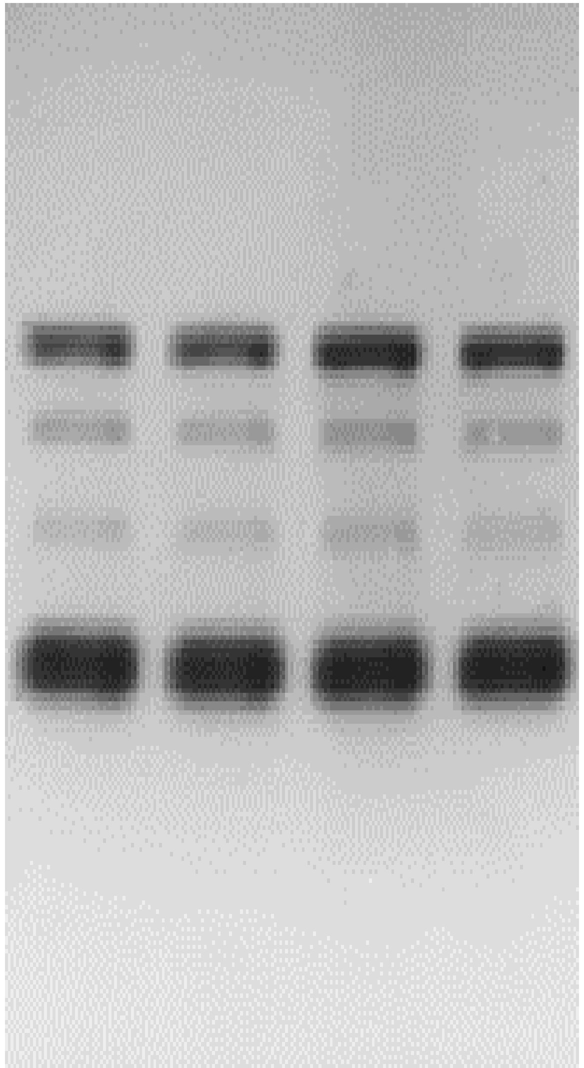

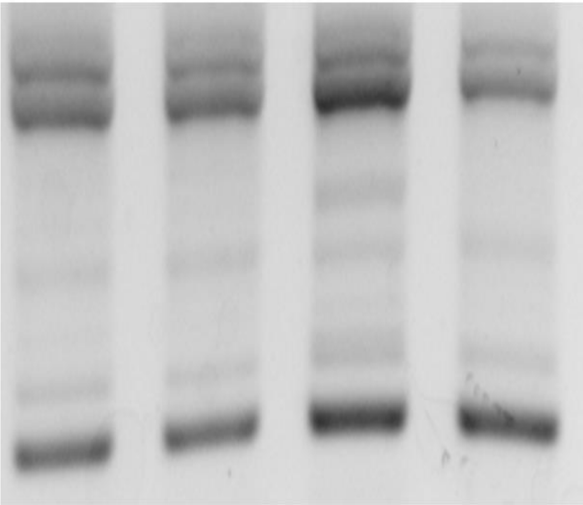

Cellular viability

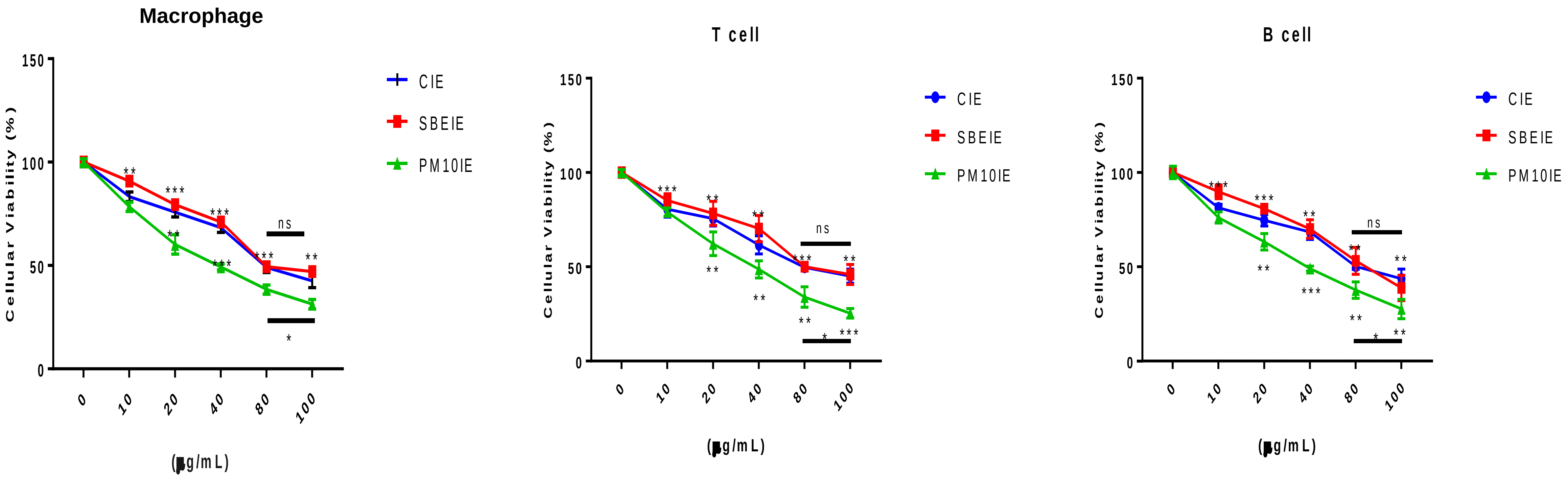

ns, not significant. * $P < 0.05$, ** $P < 0.01$, *** $P < 0.001$.

Images of Exosomal internalization in PDLSCs

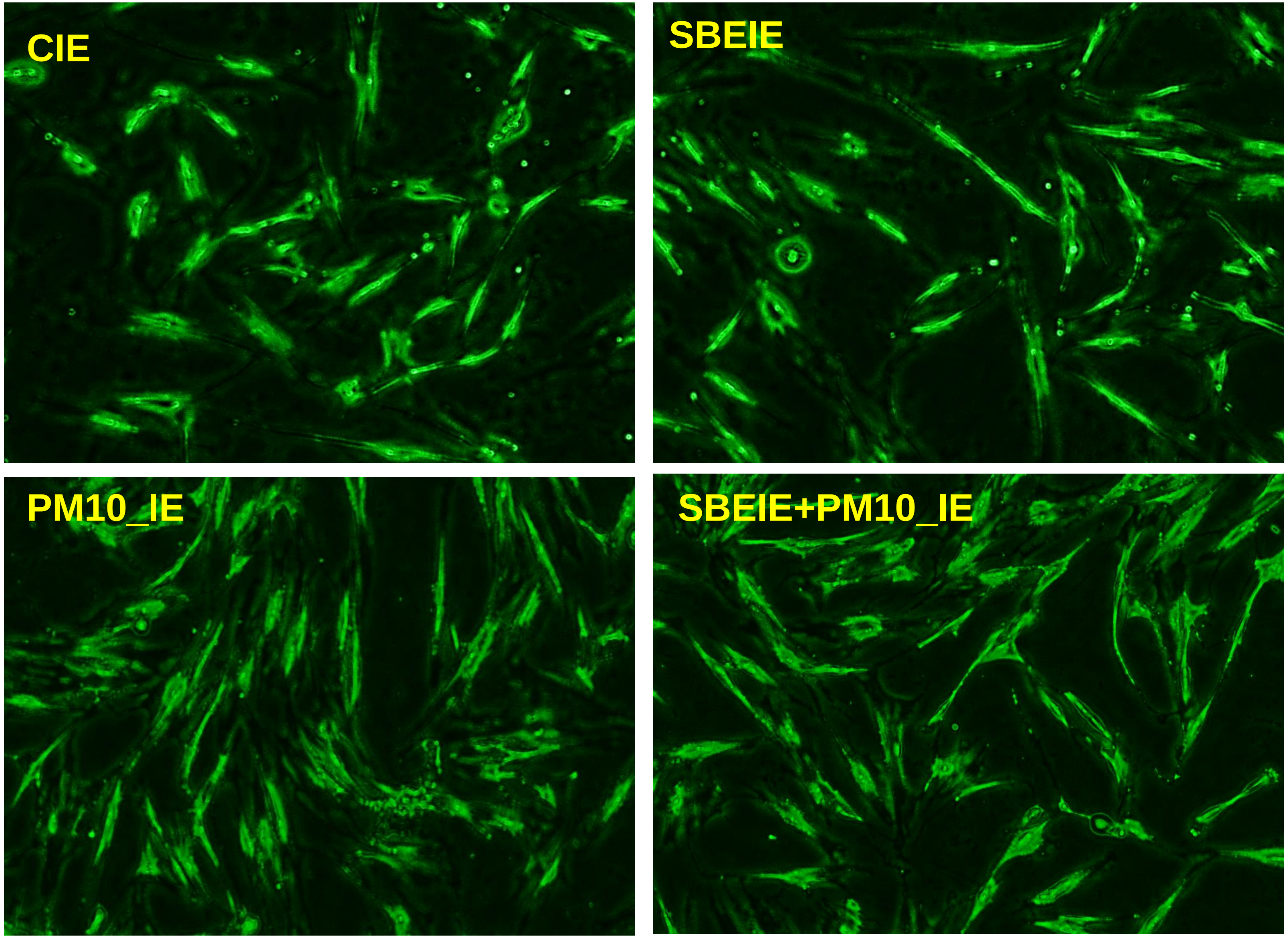

Anti-CD68 (FITC)
